# Dermoscopy in atypical phthiriais eyelash

**DOI:** 10.11604/pamj.2016.23.11.8541

**Published:** 2016-01-22

**Authors:** Krich Sanaa, Mernissi Fatima Zahra

**Affiliations:** 1 Dermatological Department, Hassan II university Hospital, Fes, Morocco

**Keywords:** Phthiriais, eyelash, dermoscopy

## Image in medicine

A 47 year-old male, with no history of pathological notables who present since 3 years chronic and severe itching eyelids pushing the patient to remove her eyelashes. Examination of the eyelids objectified a madarosis with irritation of eyelids (A). Dermoscopic examination revealed the presence of nits attached to the eyelashes (B). There was any lesions on the examination of other body areas. Based on the observation of nits at the base of the eyelashes (C) a diagnosis of phthiriasis palpebrarum was mad. Patient was treated with oral ivermectin associated to hygiene measure. Phthiriasis palpebrarum (lice infestation of eyelids), caused by the Phthirus pubis, is an unusual cause of blepharitis and conjunctivitis and may easily be overlooked because of the failure of physicians to recognize P. pubis. Dermoscopy can aid in the detection of nits and in monitoring treatment response by determining whether nits contain viable or dead nymphs or are empty (D).

**Figure 1:**
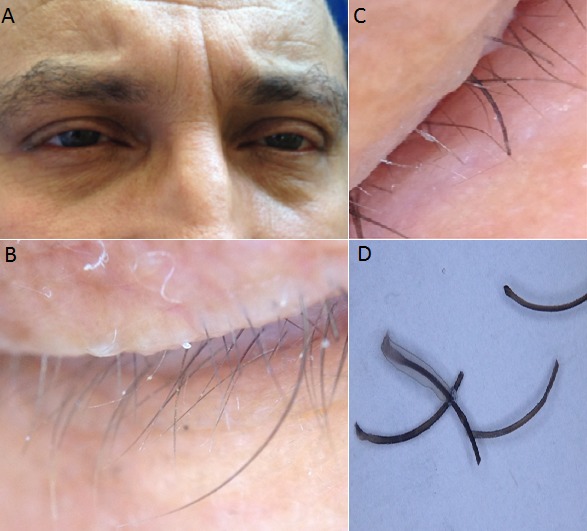
(A) madarosis with irritation of eylids; (B) presence of nits attached to the eyelashes; (C) nits at the base of the eyelashes; (D) amorphous pseudonits attached to the hair shaft

